# Protective Effects of Melatonin Against Aristolochic Acid-Induced Nephropathy in Mice

**DOI:** 10.3390/biom10010011

**Published:** 2019-12-19

**Authors:** Jung-Yeon Kim, Jaechan Leem, Eon Ju Jeon

**Affiliations:** 1 Department of Immunology, School of Medicine, Catholic University of Daegu, Daegu 42472, Korea; jy1118@cu.ac.kr; 2 Department of Internal Medicine, School of Medicine, Catholic University of Daegu, Daegu 42472, Korea

**Keywords:** melatonin, aristolochic acid, nephropathy, oxidative stress, inflammation, apoptosis, fibrosis

## Abstract

Melatonin, a pineal hormone, is well known to regulate the sleep–wake cycle. Besides, the hormone has been shown to display pleiotropic effects arising from its powerful anti-oxidant and anti-inflammatory activities. Recent studies have reported that melatonin exerts protective effects in animal models of kidney disease. However, the potential effects of melatonin on aristolochic acid (AA)-induced nephropathy (AAN) have not yet been investigated. Here, we found that the administration of melatonin ameliorated AA-induced renal dysfunction, as evidenced by decreased plasma levels of blood urea nitrogen and creatinine and histopathological abnormalities such as tubular dilatation and cast formation. The upregulation of tubular injury markers after AA injection was reversed by melatonin. Melatonin also suppressed AA-induced oxidative stress, as evidenced by the downregulation of 4-hydroxynonenal and reduced level of malondialdehyde, and modulated expression of pro-oxidant and antioxidant enzymes. In addition, p53-dependent apoptosis of tubular epithelial cells, infiltration of macrophages and CD4^+^ T cells into damaged kidneys, and renal expression of cytokines and chemokines were inhibited by melatonin. Moreover, melatonin attenuated AA-induced tubulointerstitial fibrosis through suppression of the tumor growth factor-β/Smad signaling pathway. These results suggest that melatonin might be a potential therapeutic agent for AAN.

## 1. Introduction

Aristolochic acid (AA) is a toxic compound found in medicinal plants, including genus *Aristolochia* and *Asarum*, and has been used in traditional Chinese medicine [1]. The AA-induced nephropathy (AAN) was first identified in the 1990s in Belgium. After that, new cases of AAN have been regularly reported in other countries and its true incidence is largely unknown and probably underestimated [2]. The disease is clinically characterized by rapidly progressive fibrosing interstitial nephritis that can often cause end-stage renal disease. However, there is currently no specific therapy for treating AA-induced renal injury. Many efforts have been made over the last two decades to elucidate the pathophysiology of AAN because an understanding of its mechanisms can shed light on the development of new therapeutic approaches. Accumulating evidence suggests that oxidative stress, tubular cell apoptosis, inflammation, and fibrosis are important pathogenic processes for the disease, although its detailed molecular mechanisms remain unclear [1,2].

Melatonin is a hormone synthesized mainly by the pineal gland and has been well known to regulate the sleep–wake cycle in most diurnal mammals including humans. In addition to its critical role in modulating the circadian rhythm, the hormone has been shown to exert pleiotropic effects arising from its powerful anti-oxidant and anti-inflammatory activities [3,4]. A previous study reported that administration of exogenous melatonin ameliorated renal injury and dysfunction in pinealectomized rats [5]. It has also been shown that melatonin displays protective effects in various types of acute kidney injury (AKI) [6,7,8,9,10,11] and chronic kidney disease (CKD) [12,13,14,15]. However, the effects of melatonin on AAN have not yet been investigated.

In the present study, we aim to investigate the potential effects of melatonin on AA-induced renal dysfunction and structural abnormalities and explore its underlying mechanisms.

## 2. Materials and Methods

### 2.1. Animals and Drug Treatment

Seven-week-old male C57BL/6N mice were obtained from Samtako Bio Korea (Osan, Korea) and housed at ambient temperatures (21–23 °C) under a 12 h:12 h light–dark cycle. Mice were provided free access to a normal chow diet and distilled water throughout all experiments. After a 1-week acclimatization, the mice were randomly divided into the following groups (n = 8 per group): vehicle (Veh), AA alone (AA), and AA plus melatonin (AA + MEL). The AA group was intraperitoneally injected with AA (5 mg/kg in 5% dimethyl sulfoxide (DMSO)) for 4 days [16]. The Veh group was intraperitoneally injected with an equal volume of DMSO. To investigate the effects of melatonin (Sigma-Aldrich, St. Louis, MO, USA) on AAN, the AA + MEL group was intraperitoneally injected with melatonin (20 mg/kg) [6,17] from 2 days before the first injection of AA for 16 consecutive days. The mice were sacrificed 14 days after the first injection of AA. Blood samples were collected and kidneys were rapidly isolated for subsequent analyses. All animal experiments were approved by the Institutional Animal Care and Use Committee of the Catholic University of Daegu (DCIAFCR-190517-06-Y).

### 2.2. Evaluation of Renal Function and Oxidative Stress

Blood samples were collected in ethylenediaminetetraacetic acid (EDTA)-coated tubes by cardiac puncture. Plasma was separated from whole blood using a centrifugation method. Plasma levels of blood urea nitrogen (BUN) and creatinine were analyzed using a BUN assay kit (Asan Pharmaceutical, Seoul, Korea) and a creatinine assay kit (Bioassay Systems, Hayward, CA, USA) according to the manufacturer’s instructions. Malondialdehyde (MDA) levels and reduced glutathione (GSH)/oxidized glutathione (GSSG) ratio in kidney tissues were measured using the lipid peroxidation (MDA) assay kit (Sigma-Aldrich) and the glutathione detection kit (Enzo Life Sciences, Farmingdale, NY, USA), respectively, according to the manufacturer′s instructions.

### 2.3. Histology and Immunohistochemistry (IHC)

After harvesting, kidneys were immediately fixed in 4% paraformaldehyde and then embedded in paraffin. The tissues were sectioned and stained with hematoxylin & eosin (H&E) stain, periodic acid Schiff (PAS) stain, and Masson’s trichrome stain. Images were captured using a confocal microscope (Nikon, Tokyo, Japan). Tubular injury in PAS-stained sections was assessed and scored at a ×200 magnification using 10 randomly selected fields for each kidney as follows: 0, 0%; 1, ≤10%; 2, 11–25%; 3, 26–45%; 4, 46–75%; and 5, 76–100% [18]. For IHC staining, the sections were incubated with primary antibodies against kidney injury molecule-1 (Kim-1; Abcam, Cambridge, MA, USA), neutrophil gelatinase-associated lipocalin (NGAL; Santa Cruz Biotechnology, Santa Cruz, CA, USA), 4-hydroxynonenal (4-HNE; Abcam), galectin-3 (Abcam), α-smooth muscle actin (α-SMA; Sigma-Aldrich), or collagen I (Abcam) overnight at 4 °C and then probed with a secondary antibody for 30 min at room temperature. All the sections were counterstained with hematoxylin. The percentage of positive staining was assessed in 5 randomly selected fields (×400 magnification) per each kidney using i-Solution Lite V.9.1 image analysis software (IMTechnology, Vancouver, BC, Canada). Galectin 3 positive cells or CD4 positive cells were counted in 5 randomly selected fields (×400 magnification) per each kidney.

### 2.4. TdT-Mediated dUTP Nick End Labeling (TUNEL) Staining

Apoptotic cell death was evaluated in kidney sections using the in situ cell death detection kit (Roche Diagnostics, Indianapolis, IN, USA) according to the manufacturer’s instructions. In brief, kidney sections were deparaffinized in xylene, rehydrated using descending grades of ethanol, and permeabilized for 30 min at room temperature. After washing, the sections were incubated in the TUNEL reaction mixture for 1 h at 37 °C. Nuclei were counterstained with DAPI. Images were captured using a confocal microscope (Nikon). TUNEL-stained apoptotic cells were counted in 5 randomly selected fields (×400 magnification) per each kidney.

### 2.5. Gene Expression Analysis

Total RNA was extracted from kidney tissue using the TRIzol reagent (Thermo Fisher Scientific) and then reverse transcribed into cDNA by using oligo (dT)18 primers and the AccuPower RT premix (Bioneer, Daejeon, Korea) according to the manufacturer’s instructions. Quantitative real-time reverse transcription-polymerase chain reaction (RT-PCR) was carried out using the Real-Time PCR 7500 system (Applied Biosystems, Foster city, CA, USA) and the Power SYBR Green PCR Master Mix (Applied Biosystems). Sequences of specific primers are listed in Table 1. GAPDH was used as a reference gene.

### 2.6. Western Blot Analysis

Protein samples extracted from kidney tissues were loaded onto gradient polyacrylamide gels and then transferred onto nitrocellulose membranes. The membranes were probed with specific primary antibodies as follows: anti-NGAL (Santa Cruz Biotechnology), anti-cleaved caspase-3 (Cell Signaling, Danvers, MA, USA), anti-cleaved poly(ADP-ribose) polymerase-1 (PARP-1; Cell Signaling), anti-p53 (Cell Signaling), anti-Bax (Santa Cruz Biotechnology), anti-tumor necrosis factor-α (TNF-α; Abcam), anti-interleukin-6 (IL-6; Abcam), anti-nuclear factor-κB (NF-κB) p65 (Cell Signaling), anti-p-NF-κB p65 (Cell Signaling), anti-α-SMA (Sigma-Aldrich), anti-fibronectin (Abcam), anti-transforming growth factor-β (TGF-β; R&D Systems, Minneapolis, MN, USA), anti-p-Smad2/3 (Cell Signaling), and anti-glyceraldehyde-3-phosphate dehydrogenase (GAPDH; Cell Signaling) antibody. The membranes were washed and incubated with horseradish peroxidase-conjugated secondary antibodies. Signals were detected using an enhanced chemiluminescence detection system (Thermo Fisher Scientific, Waltham, MA, USA) and analyzed using the ChemiDoc™ XRS+ Imaging System (Bio-Rad Laboratories, Hercules, CA, USA). The protein expression levels were normalized against GAPDH.

### 2.7. Statistical Analysis

Data are represented as the mean ± standard error of the mean (SEM) and analyzed using a one-way analysis of variance (ANOVA) followed by a posthoc Bonferroni’s multiple comparison test. *p* < 0.05 was considered statistically significant.

## 3. Results

### 3.1. Melatonin Ameliorated AA-Induced Structural Abnormalities and Renal Dysfunction

**Figure 1:**
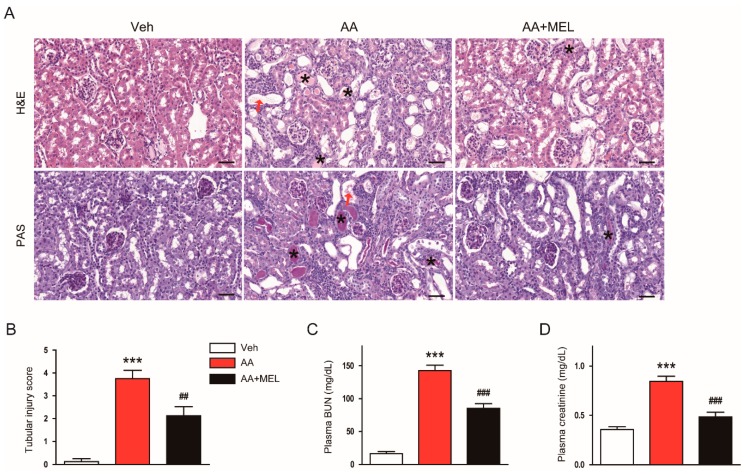
Effects of melatonin on renal function and renal histology in aristolochic acid-induced nephropathy (AAN). Mice were divided into the following groups: vehicle (Veh), AA alone (AA), and AA plus melatonin (AA + MEL). (**A**) Representative images of hematoxylin and eosin (H&E) and periodic acid Schiff (PAS) staining. Red arrows indicate tubular dilatation. Asterisks indicate cast formation. Scale bar: 50 μm. (**B**) Tubular injury score. (**C**) Plasma blood urea nitrogen (BUN). (**D**) Plasma creatinine. n = 8 per group. All data are presented as the mean ± standard error of the mean (SEM). *** *p* < 0.001 vs. Veh. ^##^ *p* < 0.01 and ^###^ *p* < 0.001 vs. AA.

It has been shown that AA induces structural abnormalities and renal dysfunction in rodents [16]. As expected, histological analyses revealed that AA-treated mice exhibited structural alterations such as tubular dilatation and cast formation compared to vehicle-treated mice (Figure 1A,B). Such effects of AA were significantly attenuated by administration of exogenous melatonin. Moreover, the hormone significantly ameliorated renal dysfunction, as evidenced by reduced levels of BUN (Figure 1C) and creatinine (Figure 1D), in AA-treated mice.

**Figure 2:**
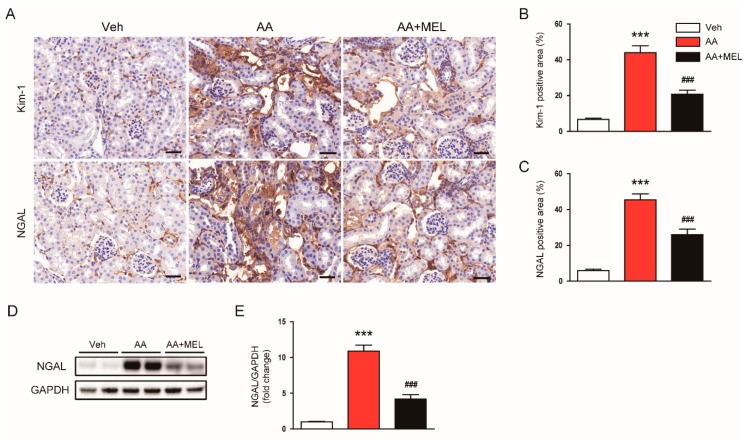
Effects of melatonin on renal expression of kidney injury molecule-1 (Kim-1) and neutrophil gelatinase-associated lipocalin (NGAL) in AAN. (**A**) Representative images of immunohistochemical staining using anti-Kim-1 or anti-NGAL antibody. Scale bar: 25 μm. The graphs show the percentage of Kim-1 positive area (**B**) or NGAL positive area (**C**) per field. (**D**) Western blot images of the expression of NGAL, and glyceraldehyde-3-phosphate dehydrogenase (GAPDH) in kidneys. (**E**) Quantification of NGAL normalized against GAPDH. n = 8 per group. All data are presented as the mean ± SEM. *** *p* < 0.001 vs. Veh. ^#^^##^ *p* < 0.001 vs. AA.

To further evaluate the effect of melatonin in tubular damage, we checked expression levels of tubular injury markers, including Kim-1 and NGAL, in kidneys. IHC staining showed that melatonin decreased elevated levels of Kim-1 (Figure 2A,B) and NGAL (Figure 2A,C) in damaged tubules of mice treated with AA. Consistently, Western blotting also confirmed that the increased protein level of NGAL after AA injection was largely reversed by melatonin (Figure 2D,E).

### 3.2. Melatonin Suppressed AA-Induced Oxidative Stress

**Figure 3:**
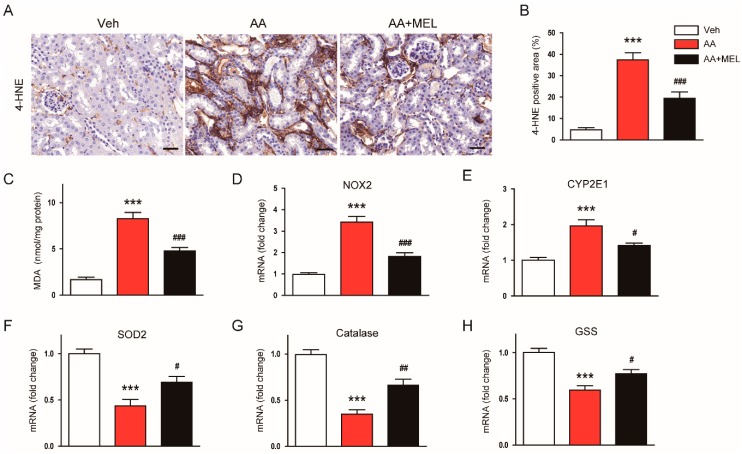
Effects of melatonin on renal oxidative damage in AAN. (**A**) Representative images of immunohistochemical staining using anti-4-hydroxynonenal (4-HNE) antibody. Scale bar: 25 μm. (**B**) Percentage of 4-HNE positive area per field. (**C**) Renal malondialdehyde (MDA) level. Real-time reverse transcription-polymerase chain reaction analysis of nicotinamide adenine dinucleotide phosphate oxidase 2 (NOX2) (**D**), cytochrome P450 2E1 (CYP2E1) (**E**), superoxide dismutase 2 (SOD2) (**F**), catalase (**G**), and glutathione synthetase (GSS) (**H**) in kidneys. n = 8 per group. All data are presented as the mean ± SEM. *** *p* < 0.001 vs. Veh. ^#^ *p* < 0.05, ^##^ *p* < 0.01, and ^###^ *p* < 0.001 vs. AA.

It has been shown that oxidative stress plays an essential role in AA-induced cytotoxicity [1,2]. Melatonin has a powerful reactive oxygen species (ROS) scavenging activity and modulates pro-oxidant and antioxidant enzymes [3]. Thus, to explore mechanisms for the protective actions of melatonin on AAN, we first examined the effect of the hormone on the renal oxidative stress in AA-treated mice. IHC staining with a monoclonal antibody directed to 4-HNE, a product of lipid peroxidation, revealed that administration of melatonin significantly reduced the 4-HNE-positive area in the kidneys of AA-treated mice (Figure 3A,B). Elevated levels of MDA, another product of lipid peroxidation, were also markedly reversed by melatonin (Figure 3C).

Nicotinamide adenine dinucleotide phosphate oxidase 2 (NOX2) is expressed in the kidney and is known to be the main source of reactive oxygen species (ROS) in the pathogenesis of AAN [19]. Cytochrome P450 2E1 (CYP2E1) is also shown to be upregulated in response to various renal insults [20,21], contributing to the generation of ROS and resultant oxidative stress. We found that mRNA levels of NOX2 (Figure 3D) and CYP2E1 (Figure 3E) in the kidneys of mice treated with AA was increased compared to vehicle-treated mice and such effects of AA were attenuated by melatonin. In addition, decreased expression of antioxidant enzymes including superoxide dismutase 2 (SOD2; Figure 3F), catalase (Figure 3G), and glutathione synthetase (GSS; Figure 3H) after AA injection were also significantly reversed by melatonin.

### 3.3. Melatonin Protected from AA-Induced Tubular Cell Apoptosis

**Figure 4:**
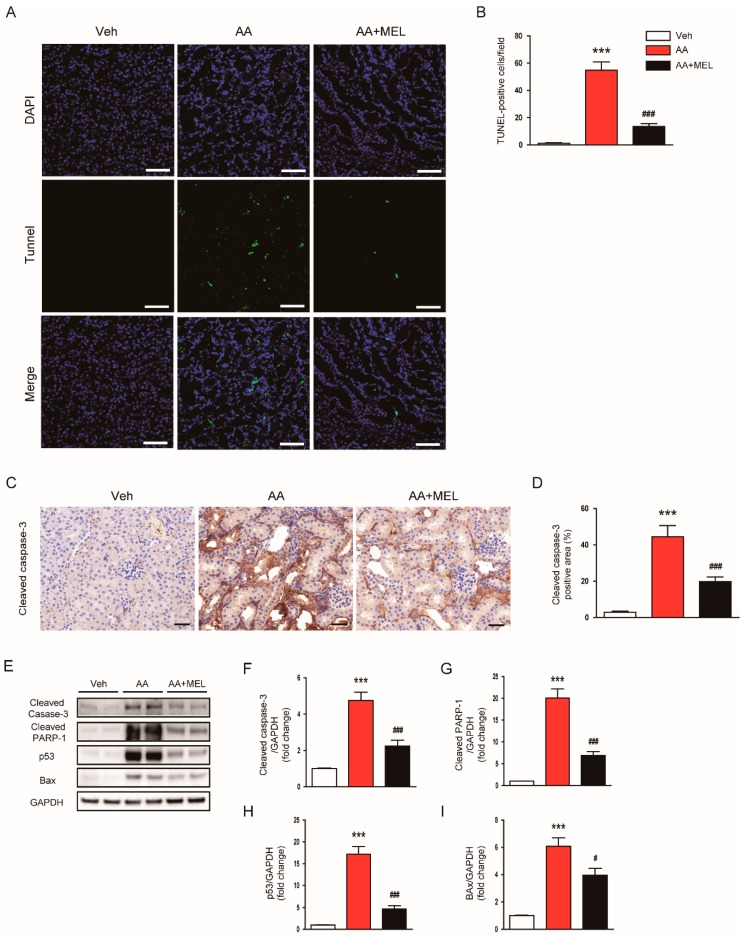
Effects of melatonin on the apoptotic death of tubular cells in AAN. (**A**) Representative images of terminal deoxynucleotidyl transferase-tediated deoxyuridine triphosphate nick end labeling (TUNEL) staining. Scale bar: 25 μm. (**B**) Number of TUNEL-stained cells. (**C**) Representative images of immunohistochemical staining using anti-cleaved caspase-3 antibody. Scale bar: 25 μm. (**D**) Percentage of cleaved caspase-3 positive area per field. (**E**) Western blot images of the expression of cleaved caspase-3, cleaved poly(ADP-ribose) polymerase-1 (PARP-1), p53, Bax, and GAPDH in kidneys. The graphs show densitometric quantification of cleaved caspase-3 (**F**), cleaved PARP-1 (**G**), p53 (**H**), and Bax (**I**) normalized against GAPDH. n = 8 per group. All data are presented as the mean ± SEM. *** *p* < 0.001 vs. Veh. ^#^ *p* < 0.05 and ^###^ *p* < 0.001 vs. AA.

Apoptosis cell death has also been implicated in direct cytotoxicity of AA on renal tubular epithelial cells [1,2]. Thus, we next investigated the effect of melatonin on AA-induced tubular cell apoptosis. We observed that an increase in TUNEL-stained cells after AA injection was significantly reduced by melatonin (Figure 4A,B). IHC staining showed that administration of melatonin largely reversed the increased expression of cleaved caspase-3 in damaged tubules of mice treated with AA (Figure 4C,D). In addition, the elevated protein levels of cleaved caspase-3 (Figure 4E,F) and cleaved PARP1 (Figure 4E,G) in the kidneys of mice treated with AA were significantly attenuated by melatonin, indicating that melatonin suppressed AA-induced apoptotic cell death. To gain further mechanistic insights into the effect of melatonin on apoptosis, we examined protein levels of p53 in the kidneys. We observed that protein expression of p53 was markedly increased in AA-treated mice compared to vehicle-treated mice (Figure 4E,H). AA-treated mice also exhibited elevated levels of Bax, a transcriptional target of p53, in the kidneys (Figure 4E,I). Interestingly, melatonin significantly suppressed the increase in protein expression of p53 and Bax in the kidneys of mice treated with AA.

### 3.4. Melatonin Attenuated AA-Induced Inflammation

**Figure 5:**
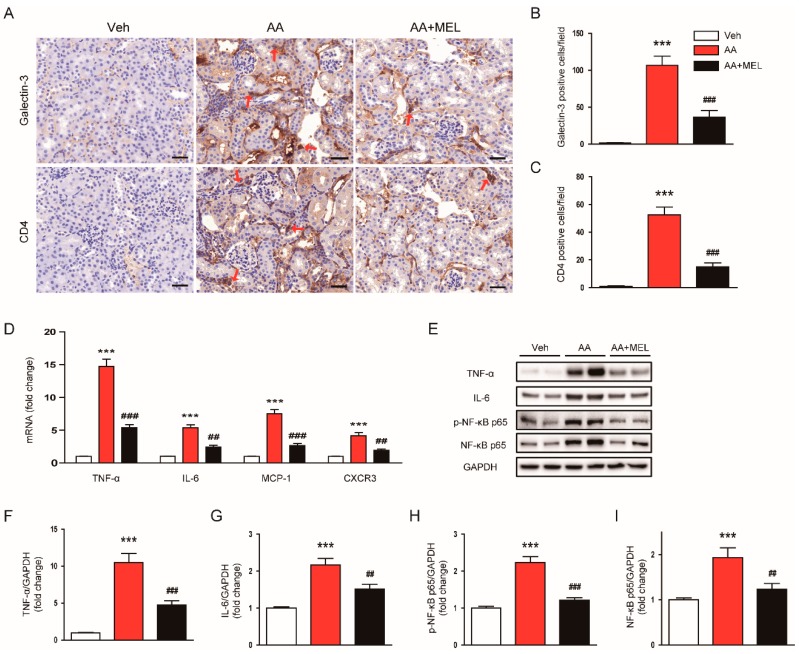
Effects of melatonin on renal inflammation in AAN. (**A**) Representative images of immunohistochemical staining using anti-galectin-3 or anti-CD4 antibody. Red arrows indicate positively stained cells. Scale bar: 25 μm. The graphs show the number of galectin-3 positive cells (**B**) or CD4 positive cells (**C**) per field. (**D**) Real-time reverse transcription-polymerase chain reaction analysis of TNF-α, IL-6, monocyte chemoattractant protein-1 (MCP-1), and C-X-C motif chemokine receptor 3 (CXCR3) in kidneys. (**E**) Western blot images of the expression of tumor necrosis factor-α (TNF-α), interleukin-6 (IL-6), p-nuclear factor-κB (NF-κB) p65, NF-κB p65, and GAPDH in kidneys. The graphs show densitometric quantification of TNF-α (**F**), IL-6 (**G**), p-NF-κB p65 (**H**), NF-κB p65 (**I**) normalized against GAPDH. n = 8 per group. All data are presented as the mean ± SEM. *** *p* < 0.001 vs. Veh. ^#^^#^ *p* < 0.01 and ^###^ *p* < 0.001 vs. AA.

Besides direct cytotoxicity, inflammation also plays a critical role in the pathophysiology of AAN [1,2]. Thus, we next investigated the effect of melatonin on AA-induced inflammatory responses. To evaluate the role of the hormone on macrophage accumulation in the kidneys after AA injection, the tissues were stained with anti-galectin-3 antibody to detect macrophages. We observed that the administration of melatonin significantly reduced the number of galectin-3-positive cells in AA-treated mice, indicating its suppressive effect on macrophage accumulation in the kidneys (Figure 5A,B). In addition, CD4^+^ T cell accumulation in the kidneys of mice treated with AA was significantly attenuated by melatonin (Figure 5A,C). Consistently, elevated mRNA levels of pro-inflammatory cytokines secreted from macrophages and T cells, including TNF–α and IL-6, and chemokines that play a critical role in attracting macrophages and T cells into the tissues, including monocyte chemoattractant protein-1 (MCP-1) and C-X-C motif chemokine receptor 3 (CXCR3), were also significantly decreased by melatonin (Figure 5D). Moreover, melatonin significantly reduced increased protein levels of TNF –α (Figure 5E,F) and IL-6 (Figure 5E,G) in the kidneys of mice treated with AA. Given that NF-κB is a critical transcription factor for modulating inflammatory response, we also evaluated the effect of melatonin on activation of the transcription factor. We found that the administration of melatonin significantly reversed the increased expression of p-NF-κB p65 (Figure 5E,H) and NF-κB p65 (Figure 5E,I) in the kidneys of mice treated with AA.

### 3.5. Melatonin Attenuated AA-Induced Fibrosis

**Figure 6:**
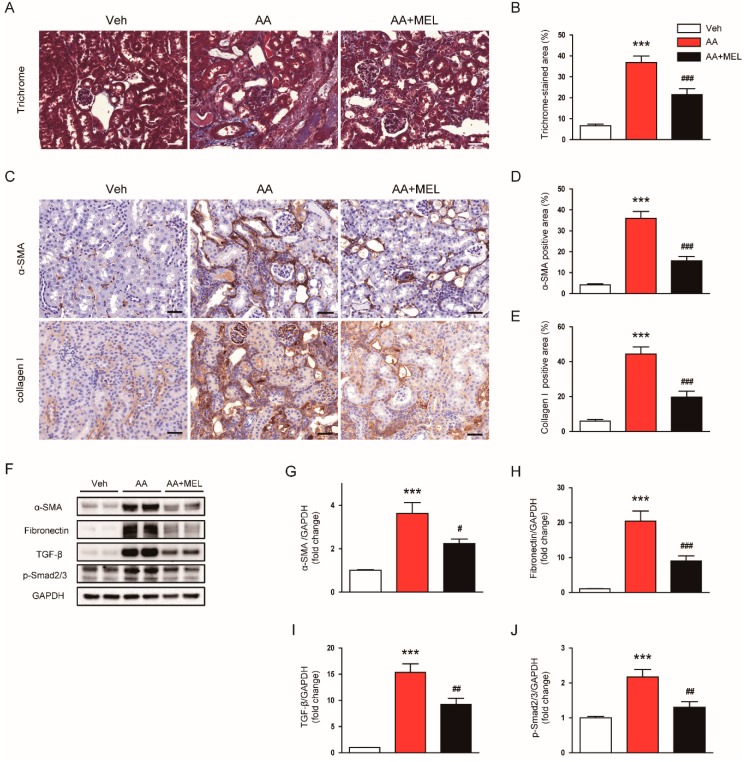
Effects of melatonin on tubuointerstitial fibrosis in AAN. (**A**) Representative images of trichrome staining. Scale bar: 25 μm (**B**) Percentage of Masson’s trichrome-stained area per field. (**C**) Representative images of immunohistochemical staining using anti-α-smooth muscle actin (α-SMA) or collagen I antibody. Scale bar: 25 μm. The graphs show the percentage of α-SMA positive area (**D**) or collagen I positive area (**E**) per field. (**F**) Western blot images of the expression of α-SMA, fibronectin, transforming growth factor-β (TGF-β), p-Smad2/3, and GAPDH in kidneys. The graphs show densitometric quantification of α-SMA (**G**), fibronectin (**H**), TGF-β (**I**), and p-Smad2/3 (**J**) normalized against GAPDH. n = 8 per group. All data are presented as the mean ± SEM. *** *p* < 0.001 vs. Veh. ^#^ *p* < 0.05, ^##^ *p* < 0.01, and ^###^ *p* < 0.001 vs. AA.

AA is known to induce tubulointerstitial fibrosis in the kidneys of rodents [1,2]. As expected, Masson’s trichrome staining showed that AA-treated mice exhibited marked tubulointerstitial fibrosis (Figure 6A,B). Interestingly, the administration of melatonin significantly reduced the area of fibrotic lesions in the kidneys of mice treated with AA. IHC staining also revealed that increased expression of α-SMA (Figure 6C,D) and collagen I (Figure 6C,E) after AA injection was significantly attenuated by melatonin. Consistently, Western blotting showed that melatonin significantly decreased protein levels of α-SMA (Figure 6F,G) and fibronectin (Figure 6F,H) in the kidneys of mice treated with AA. Moreover, increased protein expression of TGF-β, a key regulator of fibrosis, after AA injection was markedly suppressed by melatonin (Figure 6F,I). Administration of melatonin also significantly reduced Smad2/3 phosphorylation, a downstream signal of TGF-β, in the kidneys of mice treated with AA (Figure 6F,J).

## 4. Discussion

In this study, we investigated the effect of melatonin, a pineal hormone that regulates the circadian rhythm, on AAN, and explored the underlying mechanisms. We found that the administration of melatonin significantly ameliorated AA-induced renal dysfunction and structural injury. These effects of the hormone are associated with the inhibition of oxidative stress, apoptotic cell death of tubular epithelial cells, inflammation, and fibrosis. Our data demonstrated for the first time that melatonin protects the kidney from AAN through its pleiotropic activities.

AAN is a rapidly progressive fibrosing interstitial nephritis frequently accompanied by end-stage renal disease and urothelial malignancies. Since its discovery in the 1990s in Belgium, the disease has become a global health challenge [2]. However, there is no effective therapy currently available for AAN. Thus, it is clinically highly important to develop a new therapeutic approach to suppress AA-induced renal injury and retard renal function decline. In the present study, we showed that administration of melatonin effectively ameliorates acute renal failure, as evidenced by a reduction in plasma levels of BUN and creatinine and histopathological alterations such as tubular dilatation, cast formation, and interstitial fibrosis induced by AA. Previous studies have shown that severe tubular injuries were observed in the kidneys of humans [22] and rodents [23] with AAN. Indeed, we also found that increased expression of tubular injury markers, including Kim-1 and NGAL, was markedly attenuated by melatonin, indicating its protective effect on AA-induced tubular injury. Altogether, these findings suggest that melatonin protects from AA-induced renal dysfunction and structural damage in mice.

It is well known that AA-deoxyribonucleic acid (DNA) adduct formation and gene mutations play a key role in the carcinogenic role of AA [24]. However, the mechanisms for cytotoxic effects of AA remain incompletely understood. Growing evidence suggests that oxidative stress, tubular cell apoptosis, inflammation, and fibrosis are important pathogenic processes for AAN, although its detailed molecular mechanisms are still unclear [1,2]. Among them, oxidative stress is known to play an essential role in AA-induced cytotoxicity, because previous in vitro and in vivo studies have reported that AA treatment induces ROS generation and resultant oxidative injury [25,26,27]. In the present study, we performed IHC staining with a monoclonal antibody directed to 4-HNE, a product of lipid peroxidation, to detect oxidative injury. As expected, the 4-HNE-positive area was markedly increased in the kidneys of AA-treated mice compared to vehicle-treated mice. Such an effect of AA was significantly suppressed by the administration of exogenous melatonin. In addition, increased levels of MDA, another product of lipid peroxidation, and decreased GSH/GSSG ratio, an indicator of oxidative stress, after AA injection were markedly attenuated by melatonin, indicating that melatonin effectively inhibits AA-induced oxidative stress. Because NOX2 is known to be a main source of ROS in the pathogenesis of AAN [19], we next evaluated the effect of melatonin on renal expression of the enzyme. We found that increased mRNA levels of NOX2 in the kidneys of mice treated with AA were significantly attenuated by melatonin. Administration of melatonin also reversed the elevated expression of CYP2E1, another pro-oxidant enzyme, in AA-treated mice. In line with our findings, previous studies showed that CYP2E1 is upregulated in response to various renal insults [20,21], contributing to the generation of ROS and resultant oxidative stress. Besides pro-oxidant enzymes, antioxidant enzymes are also implicated in AA-induced oxidative stress. It has been shown that AA-treated animals exhibited reduced antioxidant capacity [27,28]. In the present study, we also observed the downregulation of antioxidant enzymes including SOD2, catalase, and GSS in the kidneys of mice treated with AA. These changes were significantly reversed by melatonin. Altogether, these results suggest that modulation of pro-oxidant and antioxidant enzymes by melatonin presumably contributes, at least in part, to its protective effect against AA-induced oxidative injury. In good agreement with our findings, melatonin has been shown to suppress oxidative stress and regulate pro-oxidant and antioxidant enzymes in animal models of AKI [8,9,10,11] and CKD [12,13,14]. Besides, the hormone is well known to have potent ROS-scavenging activity [3]. Thus, we cannot exclude the possibility that its direct ROS-scavenging activity is also importantly involved in the protective effects of the hormone against AA-induced oxidative injury.

In addition to the role of oxidative stress, apoptosis of tubular epithelial cells is also recognized as a critical pathogenic event in AAN [1,2]. Indeed, previous studies have reported the pro-apoptotic effects of AA on tubular epithelial cells. In the present study, we observed that AA-treated mice displayed an increased number of TUNEL-stained cells with elevated protein levels of cleaved caspase-3 and cleaved PARP1 in the kidneys. Such effects of AA were effectively suppressed by melatonin. In line with our findings, melatonin has been shown to exert anti-apoptotic effects in animal models of AKI [6,7,8]. In order to obtain further mechanistic insights into the anti-apoptotic effects of melatonin, we performed Western blot analysis to assess protein levels of p53 in the kidneys, because p53 knockout mice were resistant to AA-induced apoptosis of tubular epithelial cells [29]. We found that the administration of melatonin significantly suppressed elevated protein expression of p53 and its transcriptional target, Bax, in the kidneys of mice treated with AA. Altogether, these results suggest that melatonin inhibited AA-induced apoptosis of tubular epithelial cells mainly through suppressing p53-dependent pathway.

Besides the direct cytotoxicity of AA, inflammation also plays a critical role in the pathophysiology of AAN [1,2]. Previous studies showed massive interstitial infiltration of macrophages and T cells into the kidneys in AAN [30,31]. In the present study, we observed that AA-treated mice exhibited increased accumulation of galactin-3-positive macrophages and CD4^+^ T cells in the kidneys. Interestingly, administration of melatonin effectively prevented immune cell infiltration into the kidneys. In addition, increased expression of chemokines that play a critical role in attracting macrophages and T cells into the tissues, including MCP-1 and CXCR3, were also significantly decreased by melatonin. Because infiltrating immune cells can secret pro-inflammatory cytokines such as TNF–α and IL-6, we next examined renal levels of the cytokines. We found that melatonin significantly suppressed the elevated expression of the cytokines with inhibition of NF-κB activation in the kidneys of mice treated with AA. Consistent with these findings, we recently reported that melatonin inhibited NF-κB signaling pathway and cytokine expression in cisplatin-induced AKI [6]. Altogether, these findings suggest that melatonin effectively inhibits AA-induced inflammatory responses through modulating immune cell infiltration and activity.

Tubulointerstitial fibrosis is a final common pathway to end-stage renal disease and is an important pathological feature of AAN [1,2]. A previous study showed that AA-treated mice displayed an accumulation of vimentin- and α-SMA-positive cells with excessive production of TGF-β in the kidneys [27]. TGF-β is a key driver of fibrotic processes including fibroblast activation and epithelial-mesenchymal transition, resulting in myofibroblast accumulation [32]. Activated myofibroblasts produce and secret extracellular matrix proteins such as collagen and fibronectin. Smad signaling is the canonical TGF-β1 signaling cascade and modulate gene expression required for the fibrotic processes. Interaction of TGF-β1 with its receptor induces Smad2/3 phosphorylation and the phosphorylated proteins assemble into a complex with Smad4 [33]. Then, the complex transports to the nucleus to regulate the expression of fibrosis-related genes. In the present study, trichrome staining and IHC staining with anti-α-SMA antibody or anti-collagen I antibody clearly showed that administration of melatonin effectively suppressed AA-induced fibrosis. Increased production of TGF-β and activation of Smad2/3 along with an elevated expression of α-SMA, a myofibroblast marker, and fibronectin after AA injection was also significantly attenuated by melatonin, suggesting that the hormone ameliorated AA-induced fibrotic processes mainly through suppression of TGF-β/Smad signaling pathway. In line with our findings, previous studies have reported that melatonin ameliorated cyclosporine A- [34], carbon tetrachloride- [35], unilateral ureteral obstruction- [36], 5/6 nephrectomy- [13], or diabetes [15]-induced renal fibrosis.

## 5. Conclusions

Our data demonstrate that melatonin protects from AAN through inhibition of oxidative stress, apoptosis of tubular epithelial cells, inflammation, and fibrosis. Although further investigation will be required to elucidate a more detailed mechanism, these results suggest that melatonin might be a potential therapeutic agent for AAN.

## Figures and Tables

**Table 1:** Primers used in this study for quantitative real-time RT-PCR.

Gene	Primer Sequence (5′→3′)	Product Size (bp)
NOX2 ^1^	Forward: TCCTATGTTCCTGTACCTTTGTG Reverse: GTCCCACCTCCATCTTGAATC	143
CYP2E1 ^2^	Forward: GCATCCAAAGAGAGGCACACT Reverse: GGCTGGCCTTTGGTCTTTTT	58
SOD2 ^3^	Forward: GCTGCACCACAGCAAGCA Reverse: TCGGTGGCGTTGAGATTGT	54
Catalase	Forward: CAAGTACAACGCTGAGAAGCCTAAG Reverse: CCCTTCGCAGCCATGTG	75
GSS ^4^	Forward: TGCGGTGGTGCTACTGATTG Reverse: ACGGCACGCTGGTCAAA	60
TNF-α ^5^	Forward: GACGTGGAACTGGCAGAAGAG Reverse: CCGCCTGGAGTTCTGGAA	63
IL-6 ^6^	Forward: CCAGAGATACAAAGAAATGATGG Reverse: ACTCCAGAAGACCAGAGGAAAT	88
MCP-1 ^7^	Forward: TAAAAACCTGGATCGGAACCAA Reverse: GCATTAGCTTCAGATTTACGGGT	120
CXCR3 ^8^	Forward: CAGCCTGAACTTTGACAGAACCT Reverse: GCAGCCCCAGCAAGAAGA	65
GAPDH ^9^	Forward: ACTCCACTCACGGCAAATTC Reverse: TCTCCATGGTGGTGAAGACA	171

^1^ Nicotinamide adenine dinucleotide phosphate oxidase 2. ^2^ Cytochrome P450 2E1. ^3^ Superoxide dismutase 2. ^4^ Glutathione synthetase. ^5^ Tumor necrosis factor-α. ^6^ Interleukin-6. ^7^ Monocyte chemoattractant protein-1. ^8^ C-X-C motif chemokine receptor 3. ^9^ Glyceraldehyde-3-phosphate dehydrogenase.
